# Arsenic Activates the ER Stress-Associated Unfolded Protein Response via the Activating Transcription Factor 6 in Human Bronchial Epithelial Cells

**DOI:** 10.3390/biomedicines10050967

**Published:** 2022-04-22

**Authors:** Priya Wadgaonkar, Zhuoyue Bi, Junmei Wan, Yao Fu, Qian Zhang, Bandar Almutairy, Wenxuan Zhang, Yiran Qiu, Chitra Thakur, Maik Hüttemann, Fei Chen

**Affiliations:** 1 Department of Pharmaceutical Sciences, Eugene Applebaum College of Pharmacy and Health Sciences, Wayne State University, 259 Mack Avenue, Detroit, MI 48201, USA; priya.wadgaonkar@wayne.edu (P.W.); qian.zhang5@wayne.edu (Q.Z.); almutairy@wayne.edu (B.A.); 2 Stony Brook Cancer Center, Renaissance School of Medicine, Stony Brook University, Lauterbur Drive, Stony Brook, NY 11794, USA; zhouyue.bi@stonybrookmedicine.edu (Z.B.); yao.fu.1@stonybrook.edu (Y.F.); wanxuan.zhang@stonybrook.edu (W.Z.); yiran.qiu@stonybrookmedicine.edu (Y.Q.); chitra.thakur@stonybrookmedicine.edu (C.T.); 3 Center for Molecular Medicine and Genetics, School of Medicine, Wayne State University, 540 E. Canfield Avenue, Detroit, MI 48201, USA; am4472@wayne.edu (J.W.); mhuttema@wayne.edu (M.H.); 4 College of Pharmacy, Al-Dawadmi Campus, Shaqra University, Riyadh P.O. Box 11961, Saudi Arabia; 5 Department of Pathology, Renaissance School of Medicine, Stony Brook University, 101, Nicolls Road, Stony Brook, NY 11794, USA

**Keywords:** arsenic, ER stress, UPR, metabolism, ATF6, Nrf2

## Abstract

Arsenic is a well-known human carcinogen associated with a number of cancers, including lung cancers. We have previously shown that long-term exposure to an environmentally relevant concentration of inorganic arsenic (As^3+^) leads to the malignant transformation of the BEAS2B cells, and some of the transformed cells show cancer stem-like features (CSCs) with a significant upregulation of glycolysis and downregulation of mitochondrial oxidative phosphorylation. In the present report, we investigate the short-term effect of As^3+^ on the endoplasmic reticulum (ER) stress response—the “unfolded protein response (UPR)” and metabolism in human bronchial epithelial cell line BEAS-2B cells. Treatment of the cells with inorganic As^3+^ upregulated both glycolysis and mitochondrial respiration. Analysis of ER UPR signaling pathway using a real-time human UPR array revealed that As^3+^ induced a significant up-regulation of some UPR genes, including ATF6, CEBPB, MAPK10, Hsp70, and UBE2G2. Additional tests confirmed that the induction of ATF6, ATF6B and UBE2G2 mRNAs and/or proteins by As^3+^ is dose dependent. Chromosome immunoprecipitation and global sequencing indicated a critical role of Nrf2 in mediating As^3+^-induced expression of these UPR genes. In summary, our data suggest that As^3+^ is able to regulate the ER stress response, possibly through activating the ATF6 signaling.

## 1. Introduction

Arsenic is a well-known toxicant found ubiquitously in the environment. It has been used since ancient times both as a curative and poison. Typical uses of arsenic are in agricultural pesticides, pigmentation, metallurgy, medications, and pyrotechnics. Various epidemiological, case–control and experimental studies have linked arsenic exposure to the development of lung, skin, bladder, kidney, and liver cancers in humans. Hence, to protect human health, the International Agency for Research on Cancer (IARC) classified arsenic and its compounds into group IA carcinogens. However, despite the prohibition on the use of arsenic in commodities, approximately 200 million people worldwide are still being exposed to arsenic via drinking water, food, and air [1,2]. Drinking water contamination is the primary source of arsenic exposure worldwide. The current US Environmental Protection Agency (US EPA) and World Health Organization (WHO) arsenic standard in drinking water is 10 ppb (<0.1 μM, 10 μg/L). However, there are areas in the US, India, Bangladesh, China, Chile, Mongolia, Mexico, and Argentina where more than 50 ppb of arsenic is found in drinking water [1,2,3]. Arsenic is known to induce cancer via various mechanisms, such as oxidative stress caused by the generation of reactive oxygen species (ROS), epigenetic alterations by regulating microRNAs, DNA and histone tail modifications, and several intracellular stress signaling pathways leading to the activation of JNK, STAT3, NF-κB, DNA damage response, and immune surveillance systems. Thus, interpreting the carcinogenic mechanisms of arsenic is the key to develop anti-cancer therapeutics.

Cancer-related mortality rates, especially of the lung, bronchus, and trachea cancers, hold the sixth position in the world’s top 10 list of leading causes of death, according to the World Health Organization (WHO) analysis of 2019. Lung cancer is the most common cancer worldwide and accounts for a significant portion of cancer-related deaths. Lung cancer can be categorized as non-small and small cell lung cancer. Non-small cell lung cancer (NSCLC) forms about 80% of the total lung cancer cases, which can be further categorized into adenocarcinoma (40%), squamous cell carcinoma (25%) and large cell carcinoma (10%). Despite advances in cancer therapy, relapse is still a major cause of cancer mortality [4,5]. This relapse has been partially attributed to a subpopulation of cells called the tumor-initiating cells or cancer stem-like cells (CSCs). The CSCs can develop from differentiated cells or bulk cancer cells. Like the normal stem cells, CSCs can self-renew and differentiate into new cell types [6]. CSCs contribute to sustained tumor growth, heterogeneity, metastasis, and chemotherapy resistance [7].

Previous studies from our lab have demonstrated that long-term treatment with an environmentally relevant concentration of inorganic arsenic (0.125–0.25 μM As^3+^) leads to the malignant transformation of the human bronchial epithelial BEAS-2B cells. The transformed cells showed malignant characteristics such as asymmetric division and formation of tumorspheres in tumor formation media. These cells also showed higher expression of stemness factors such as Oct4, Sox2, Nanog, c-myc, etc., compared to the non-transformed cells. Henceforth, we refer to these cells as the arsenic-induced CSCs or CSCs. By subcutaneous inoculation of 10,000 control cells and the As^3+^-induced CSCs, respectively, into athymic nude mice, only CSCs, but not the control cells, could form fast-growing tumors [8,9].

Meanwhile, a metabolic change was observed in the As^3+^-induced CSCs compared to non-CSCs cells. Transcriptomics data revealed an upregulation of the glycolytic genes and metabolites. In contrast, a significant downregulation of mitochondrial oxidative phosphorylation (OXPHOS) genes was observed [7]. Interestingly, a reduced expression of ER stress and autophagy genes in the As^3+^-induced CSCs was also observed, which indicated a possible connection between ER stress signaling and energy metabolism during the generation of CSCs induced by As^3+^.

ER stress-activated unfolded protein response (UPR) and autophagy are essential in maintaining mitochondrial dynamics, such as mitochondrial fusion, fission, and metabolism, and recycling misfolded proteins and organelles to provide alternative fuel during stress/nutrient deprivation conditions. There are three master regulatory arms of the UPR signaling: protein kinase RNA-like ER kinase (PERK), inositol-requiring enzyme 1 (IRE1) and activating transcription factor 6 (ATF6). The ATF6 has two genes at different genomic locations. The gene encodes ATF6A, also named ATF6, is at the gene locus of chromosome 1 (q23.3), whereas the gene of ATF6B is at a locus of chromosome 6 (p21.32). Under certain stress conditions, an intimate connection between the ER, mitochondrion and autophagy has been well-established. Impairment in either one of them directly affects the functioning of the other. Thus, it becomes essential to investigate whether As^3+^ affects the interaction among ER, mitochondrion, and autophagy during the process of As^3+^-induced generation of the CSCs [10,11,12]. Meanwhile, induction of the CSCs by As^3+^ is made by a relatively long-term treatment scheme, in which cells undergo positive and negative selection, adaptation and repopulation. Thus, recognizing the initiation cellular response, or short-term treatment of the cells with As^3+^, will be critical to determine how ER stress signaling contributes to the As^3+^-induced carcinogenesis. Answering these questions, accordingly, may also help us to understand if UPR, metabolism and autophagy play a role in the malignant transformation of the BEAS2B cells or maintenance of the stemness features of the As^3+^-induced CSCs.

## 2. Materials and Methods

### 2.1. Cell Culture

The human bronchial epithelial cell line BEAS-2B was purchased from American Type Culture Collection (ATCC, Manassas, VA, USA). The BEAS-2B cells were cultured in Dulbecco’s Modified Eagle’s Medium-High Glucose (Sigma-Aldrich #D5796) with added 5% Fetal Bovine Serum, 1% L-Glutamine and 1% Penicillin/Streptomycin in a humidified incubator at 37° C and 5% CO_2_. The cells were starved with serum-free medium (overnight), followed by treatment of the cells with various concentrations of As^3+^ [arsenic (III) chloride, Sigma-Aldrich, St. Louis, MO, USA] for the indicated times. For long-term treatment, BEAS-2B cells were exposed to 0.125–0.25 μM As^3+^ for six months. These cells are referred to as transformed BEAS-2B cells.

### 2.2. Human Unfolded Protein Response Qiagen RT^2^ Profiler PCR Array

Total RNA was isolated from control BEAS-2B cells, or the cells were treated with As^3+^ using Qiagen RNeasy plus mini kit (Qiagen, Germantown, MD, USA). The RNA quality was determined by agarose gel electrophoresis, and concentration was calculated using the Thermofisher nanodrop spectrophotometer. RNA was reverse transcribed to cDNA using a High-Capacity RNA-to-cDNA kit (Applied Biosystems, Waltham, MA, USA) according to the manufacturer’s instructions. The cDNA and SYBR-Green qPCR Master mix (Qiagen Cat. no. 330529) were added to the Qiagen Human UPR array plate (Qiagen, Cat. no. PAHS-089Z) to detect changes in gene expression in the UPR with or without As^3+^ treatment.

### 2.3. Regular Reverse Transcription Polymerase Chain Reaction (PCR)

Total RNA was isolated from As^3+^-treated and control BEAS-2B cells prepared using Qiagen RNeasy plus mini kit according to the manufacturer’s protocol. Reverse transcription was performed to convert RNA to cDNA using High-Capacity RNA-to-cDNA kit (Applied Biosystems) according to the manufacturer’s instructions. ATF6 and UBE2G2 primers were designed using the NCBI primer blast. GAPDH was used as a loading control. RT-PCR primer sequences for ATF6α (exon 16) are as follows: forward primer, 5′-GAAGCTTATGGCAGAGATGCAC-3′; reverse primer, 5′-CAGTGCTTTCCAAATAGATGGGTA-3′. For UBE2G2 (exon 4), they are: forward primer, 5′-CATTTGTCAATTGTGGTCGACGTT-3′; reverse primer, 5′-AATACCACCATGCTTACTTGGCT-3′. Primer sequences for GAPDH are as follows: sense primer, 5′-CTGAACGGGAAGCTGGCATGGCCTTCC-3′; anti-sense primer, 5′-CATGAGGTCCACCTGTTGCTGTAGCC-3′. PCR products were run on 1% agarose gels with DNA ladders. Samples without cDNA or RT templates served as negative controls.

### 2.4. Western Blotting

BEAS-2B cells were seeded in a 6-well plate (3.0 × 10^5^ cells per well) or 10 cm dish (1.0 × 10^6^ cells per plate) and treated with various concentrations of As^3+^ for the indicated times. Cells were lysed by 1 × RIPA lysis buffer (Cell signaling) supplemented with protease and phosphatase inhibitors cocktail (Roche, Indianapolis, IN, USA) and 1 mM PMSF. Cell lysates were homogenized by sonication, and insoluble debris was removed through centrifugation of 12,500× *g* for 15 min at 4 °C. The protein concentrations were then determined using Pierce BCA Protein Assay Kit (Thermo Scientific, Rockford, IL, USA). The protein samples were prepared using 4 × LDS sample buffer (Invitrogen, Carlsbad, CA, USA) with dithiothreitol (final concentration of 200 mM) and were denatured by boiling at 95 °C for 5 min before separation by SDS-PAGE gel. Separated samples were then transferred onto PVDF membrane (Invitrogen) and blocked with 5% non-fat milk diluted in 1 × TBST for 1 h at room temperature followed by a quick TBST rinse. The membranes were then incubated with the indicated primary antibodies overnight at 4 °C. The membranes were further washed thrice, each wash of TBST for a period of 10 min. The membranes were further incubated at room temperature with the corresponding HRP-linked secondary antibodies for 1 h, followed by TBST wash thrice. ECL substrates (Thermo Scientific, Millipore and Westpico Plus, Waltham, MA, USA) were used to visualize the signals. Primary antibodies against p-CEBPβ Threonine 235 (3084S), SCAP (13102s), GAPDH and HRP-linked rat, mouse and rabbit IgG were purchased from Cell Signaling technology. Antibodies against ATF6 and ATF6B were purchased from Biolegend for Western blot. UBE2G2 (ab235790), HSPA1B/Hsp70 (ab231637), anti-htrA4 antibody-catalytic domain (ab65915), anti-ERN2 antibody (ab135795) and CEBPβ primary antibodies (ab32358) were purchased from Abcam.

### 2.5. Seahorse Bioanalyzer for Cell Energy Phenotype Test

The Seahorse XF24 Extracellular Flux Analyzer was used to obtain real-time measurements of extracellular acidification rates (ECAR) and oxygen consumption rate (OCR) in BEAS-2B cells. Cells (1.0 × 10^4^) were plated in a 24-well Agilent Seahorse XF24 plate in DMEM media. The plate was coated with 0.1% gelatin prior to cell seeding to improve cell adhesion. The extracellular flux assay kit cartridge was hydrated with XF calibrant/well. The hydrated cartridge was incubated overnight in the CO_2_-free incubator prior to use. After the cells reached 70–80% confluency, the media was changed to serum-free DMEM (overnight). The cells were treated with the indicated doses of arsenic. The media was changed to Seahorse media with appropriate supplements and arsenic 45 min–1 h prior to ECAR and OCR measurements.

### 2.6. Immunohistochemistry

Lung adenocarcinoma tissue microarray slide LC10013c (lung cancer and matched adjacent normal lung tissue array) was purchased from US Biomax, Inc. (Rockville, MD, USA). It was processed for immunohistochemical staining using ATF6 and ATF6B antibodies (Novus biologicals, Littleton, CO, USA). Paraffin-embedded tissue sections were deparaffinized with xylene and hydrated in a series of alcohol gradients. To quench endogenous peroxidase activity, slides were incubated with 1.5 to 3% H_2_O_2_ in PBS for 20 min at room temperature. Heat-mediated antigen retrieval was performed by boiling tissue sections in citrate buffer with pH 6.0 for 20 min in a microwave. To block nonspecific binding of immunoglobulin, slides were incubated with a solution containing 5% goat serum, 0.2% Triton X-100 in PBS for 2 h at room temperature, followed by incubation with primary antibodies against ATF6 and ATF6B (1:50) overnight at 4  °C. Goat anti-mouse and goat anti-rabbit biotinylated secondary antibodies were subsequently applied at 1:100 dilution and incubated for 2 h at room temperature. Slides were then incubated with ABC reagent (Vectastatin Elite ABC kit, Vector Laboratories Inc., Burlingame, CA, USA) for 45 minutes at room temperature. The chromogen was developed with diaminobenzidine (DAB). Slides were counterstained with hematoxylin (Sigma-Aldrich, St. Louis, MO, USA) and mounted with Entellan^®^ (Electron Microscopy Sciences, Hatfield, PA, USA). All incubation steps were carried out in a humidified chamber, and all washing steps were performed with 1 × PBS. Images were captured under a bright field of a Nikon Eclipse Ti-S Inverted microscope (Mager Scientific, Dexter, MI, USA).

### 2.7. Kaplan–Meier Survival Analysis

A Kaplan–Meier survival analysis was carried out using the Kaplan–Meier plotter (lung cancer) from mRNA database [13], which contains overall survival (OS) of 1925 lung cancer patients, using the option of the best perform threshold as a cutoff. The probe IDs are indicated on the top of each panel in the figures. Survival curves resulting in *p* values of <0.05 were considered statistically significantly. The gene expression of ATF6 and ATF6B in human lung cancer was determined by using TCGA platforms that contain genomic and transcriptiomic data of the lung cancer patient databases. The differential expression of ATF6 and ATF6B in normal lungs and lung tumors was calculated by TNMplot [14].

### 2.8. Chromatin Immunoprecipitation with Global Parallel DNA Sequencing (ChIP-seq)

ChIP-seq for Nrf2 and HIF1a was performed as reported previously [15]. Briefly, BEAS-2B cells were seeded in 10 cm dishes and treated with 1 mM As^3+^ or without treatment for 6 h. At the end of culture, approximately 10 million control cells and the As^3+^-treated cells were fixed using formaldehyde solution. The fixation was then stopped by adding glycine solution. The cell pellet was washed twice with 1× PBS-Igepal and snap-frozen in dry ice. The cells were then subjected to immunoprecipitation using ChIP-grade antibodies against Nrf2 and HIF1α from Active Motif (Carlsbad, CA, USA). The procedures of ChIP, preparation of input and control DNA, DNA sequencing, and data analysis were performed as what we had recently reported [16]. The sequene tags were aligned to the reference genome hg19 using the Burrows–Wheeler Aligner (BWA) algorithm with default settings. The enrichment data of Nrf2 and HIF1a were visualized using University of California Santa Cruz (UCSC) genome browser. All ChIP-seq data can be accessed at https://www.ncbi.nih.gov/geo/query/acc.cgi?acc=GSE145834 (accessed on 25 February 2020).

### 2.9. Statistical Analysis

All cell culture experiments were performed independently in triplicate at a minimum (unless otherwise indicated). Western blot images were analyzed using NIH ImageJ software. One-way Anova with 95% confidence interval followed by Tukey’s post hoc test was performed using IBM SPSS statistical software for Western blots and Seahorse cell phenotype data. Wilcoxon signed-rank tests and Kruskal–Wallis H tests were used for immunohistochemistry data. Figures were prepared using GraphPad Prism 5 and plotted as mean values with SEM. A *p*-value of  < 0.05 was considered statistically significant.

## 3. Results

### 3.1. Effect of Short-Term As^3+^ Treatment on the ER Stress Activated UPR

**Figure 1:**
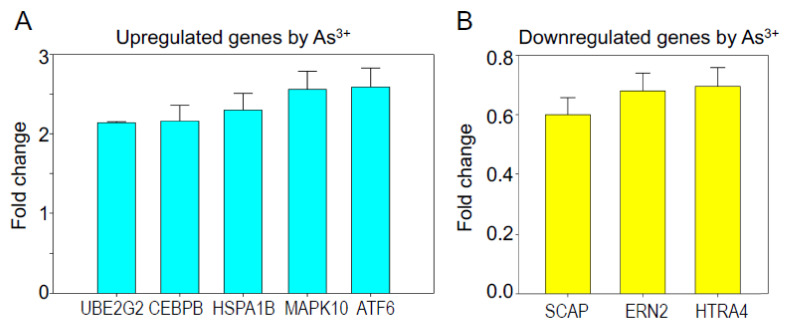
Regulation of ER stress activated unfolded protein response (UPR) by As^3+^ (**A**). As^3+^ upregulated UPR genes ATF6, CEBPB, HSPA1B, MAPK10, UBE2G2 in BEAS2B cells. Data are fold-changes of these genes in UPR array for the As^3+^-treated cells relative to the control cells. Only the genes that had more than 2-fold induction by a treatment of the cells with 2 μM As^3+^ for 6 h are shown (**B**). Downregulated UPR genes, ERN2, HTRA4, SCAP, by As^3+^. Only the genes with more than 1.2-fold downregulation are shown.

Previous studies from our lab have demonstrated that long-term As^3+^ treatment leads to the malignant transformation of the human bronchial epithelial BEAS-2B cells. Some of these cells acquire cancer stem-like (CSCs) features with a metabolic shift from mitochondrial TCA cycle to glycolysis. We also detected a significant downregulation of the ER stress response and autophagy genes in these As^3+^-induced CSCs [7,9]. To examine the short-term effect of As^3+^ on the ER stress-associated UPR, we treated BEAS-2B cells with 2 μM As^3+^ for 6 h. We had previously shown that this treatment condition did not provoke cell death or toxicity (data not shown). A real-time RT^2^ profiler human UPR PCR array was used, which contained 84 gene primers related to UPR. The genes of activating transcription factor 6 (ATF6), CCAAT/enhancer binding protein beta (C/EBPβ, CEBPB), heat shock 70 kDa protein 1B (HSPA1B), mitogen-activated protein kinase 10 (MAPK10), and ubiquitin-conjugating enzyme E2G2 (UBE2G2) were significantly induced by As^3+^ (Figure 1A). Three genes, including endoplasmic to nucleus signaling 2 (ERN2), HtrA serine peptidase 4 (HTRA4) and SREBF chaperone (SCAP), were downregulated to 1.4- to 1.8-fold in response to As^3+^ (Figure 1B). However, the ERN2, HTRA4, and SCAP protein levels were not changed in the cells treated with As^3+^ (data not shown).

The above upregulated genes ATF6, CEBPB, HSPA1B (Hsp70), MAPK10 (JNK3), and UBE2G2 are involved in the signaling pathways of the ER stress activated UPR. ATF6 (90 kDa) is one of the three UPR sensors and a basic leucine zipper transcription factor activated under ER stress. It is cleaved by serine proteases in the Golgi apparatus to release a cleaved form of ATF6 (50 kDa). Cleaved form of ATF6 translocates to the nucleus to induce UPR genes (X-box binding protein, Grp78) through activation of ER stress element (ERSE) to resolve ER stress [16,17]. CEBPB is a transcription factor that is involved in the regulation of genes that play an important role in immune and inflammatory responses. Under metabolic stress, the UPR pathway through PERK/eIF2α can stimulate CEBPB expression promoting lipogenesis [18]. HSPA1B is a heat shock protein that along with other members of its family, stabilizes proteins against aggregation and mediates folding of newly translated proteins. It is also involved in the ubiquitin–proteosome pathway. MAPK10 is a member of the MAP kinase family that is activated via the IRE1α-TRAF2 UPR signaling pathway. UBE2G2 belongs to family of ubiquitin-conjugating enzymes which catalyze the transfer of ubiquitin from ubiquitin-activating E1 enzymes to ubiquitin-conjugating E2 enzymes. This protein is part of the ER-associated degradation (ERAD) pathway of the UPR which degrades misfolded or improperly folded proteins in the cytoplasm [19].

### 3.2. As^3+^-Induced UBE2G2 and ATF6 Is Dose-Dependent

**Figure 2:**
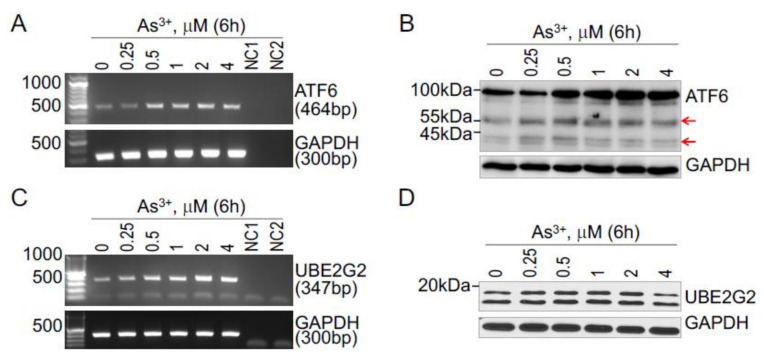
Dose-dependent induction of ATF6 and UBE2G2 by As^3+^ (**A**,**B**). Dose-dependent induction of ATF6 mRNA (**A**) and protein (**B**) by As^3+^ in BEAS-2B cells (**C**,**D**). Dose-dependent induction of UBE2G2 mRNA (**A**) and protein (**B**) by As^3+^ in BEAS-2B cells. The cells were treated with the indicated concentrations of As^3+^ for 6h.

To additionally confirm the inducibility of these ER stress-activated UPR genes by As^3+^, we next treated the BEAS-2B cells with 0, 0.25, 0.5, 1, 2, and 4 μM As^+3^ for 6 or 8 h, followed by regular reverse transcription-PCR analysis for ATF6 and UBE2G2 (Figure 2A,C). Negative control 1 (NC1) represents a reverse transcriptase-free cDNA master mix, and negative control 2 (NC2) represents the use of nuclease-free water instead of cDNA in PCR. There is a clear dose-dependent induction of ATF6 and UBE2G2 mRNAs by As^3+^ (Figure 2). To further confirm such an inducibility of As^3+^ on these two genes, the cells from such treatments were also subjected to Western blotting. Again, As^3+^ is able to induce protein accumulation of ATF6 in a dose-dependent manner (Figure 2B). It is known that ATF6 protein is proteolytically cleaved during ER stress response. The N-terminus of ATF6 has a transcriptional activation domain (TAD), whereas C-terminus has a basic, leucine-zipper (Leu-Zip) and ER transmembrane (ERTM) domains. Upon ER stress, the serine proteases in Golgi apparatus cleave the ERTM domains of ATF6 (378–398aa) to release the activated cleaved N-ATF6 transcription factor. A cleaved form of the ATF6 protein indicates that the UPR has been activated [20]. Indeed, we observed a cleaved form for ATF6 at 55 and 45 kDa, which are also roughly in an As^3+^-dose dependent manner (Figure 2B, pointed by arrows). For the UBE2G2 protein, although the induction is marginal, there is a notable induction by As^3+^ at concentrations between 0.25 to 2 mM (Figure 2D).

### 3.3. As^3+^ Induces ATF6B

**Figure 3:**
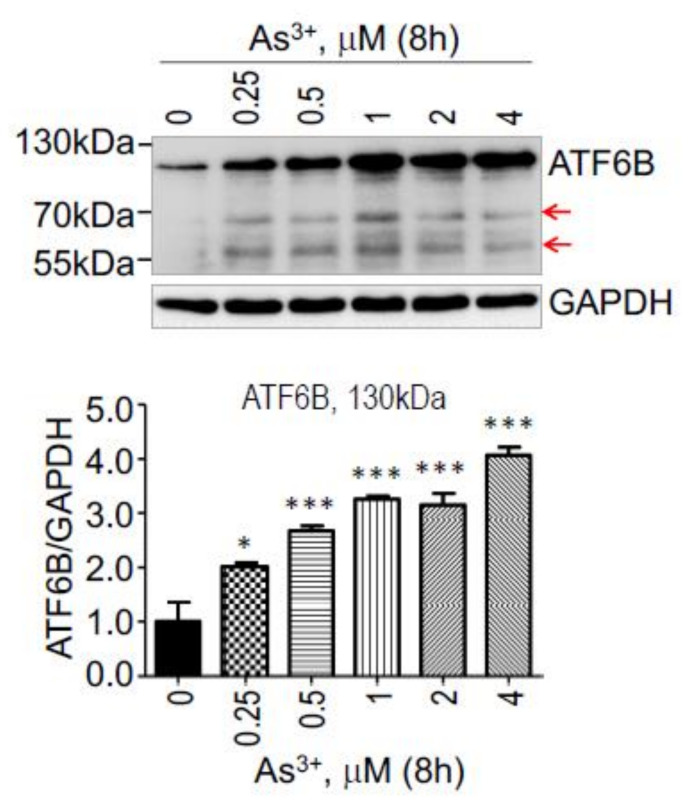
Induction of ATF6B by As^3+^ is dose dependent. The cells were treated with the indicated concentrations of As^3+^ for 8 h. Bottom panel show average of the ATF6B proteins of three independent experiments. * *p* < 0.05; *** *p* < 0.001.

It is known that ATF6 has two isoforms encoded by two different genes. The gene for the bone file ATF6, also named ATF6A, is located at the genomic region of chromosome 1 (chr1) q23.3, whereas the gene for ATF6B is positioned at chr6 p21.32. The ATF6 and ATF6B can form heterodimers to bind to the ER stress response element for the transcription of UPR genes. Mice with genetic deficiency of ATF6 or ATF6B do not elicit major phenotype, whereas the dual deficiency is lethal, suggesting equal or similar importance of ATF6 and ATF6B for the normality of the cells [21]. Since the potency of As^3+^ on the induction of ATF6 was observed, we also checked the effect of As^3+^ on ATF6B. A dose-dependent induction of ATF6B by As^3+^ was also detected. Although not well-studied, As^3+^ treatment also promoted generation of the proteolytic products of ATF6B at molecular weight around 70 and 55 kDa (Figure 3).

### 3.4. Nrf2 Dependency of the As^3+^-Induced ATF6 and ATF6B

**Figure 4:**
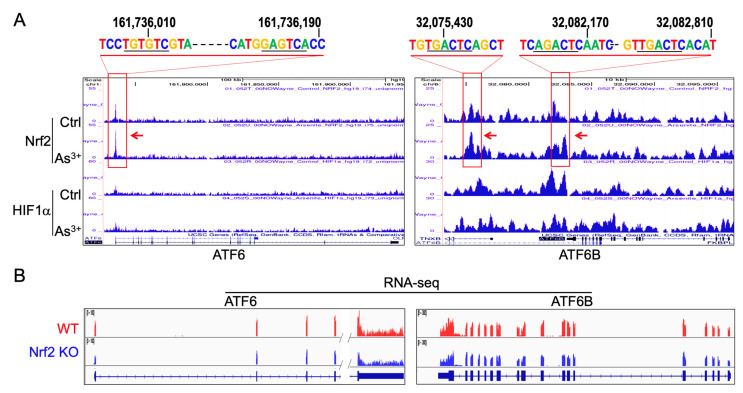
ChIP-seq analysis of Nrf2 dependency for the As^3+^-induced ATF6 and ATF6B (**A**). ChIP-seq shows an enhanced enrichment of Nrf2 (pointed by red arrows) on the promoter region of ATF6 and downstream of ATF6B gene in the cells treated with 1 mM As^3+^ for 6h. The corresponding Nrf2-binding elements for each of these Nrf2 peaks are indicated on top of the panels. Numbers above the Nrf2 motifs are genomic positions of nucleotides in human genome hg19 (**B**). RNA-seq revealed reduced expression of ATF6 and ATF6B in the Nrf2 knockout BEAS-2B cells.

To understand how As^3+^ regulates the expression of ATF6 proteins, we retrieved our ChIP-seq data from recent studies for transcription factors Nrf2 and HIF1a in the control BEAS-2B cells and the BEAS-2B cells treated with 1 mM Aa^3+^ for 6 h [22]. Visualization of the gene loci for both ATF6 and ATF6B in genome browser revealed that As^3+^ induced a substantial enhanced enrichment of Nrf2 in the gene promoter of ATF6 (left panel Figure 4A) and downstream of ATF6B (right panel of Figure 4A). There is no significant difference in the enrichment of HIF1a in both ATF6 and ATF6B between control and As^3+^-treated cells. We previously had shown that the conserved Nrf2-binding element contains a core sequence motif TGAGTC or TGACTC [22]. Manual inspection of the Nrf2 peak region of the ATF6 gene showed that there are two less-conserved Nrf2 binding motifs (indicated on the top of the ATF6 panel in Figure 4A). For ATF6B, there are two major Nrf2 enrichment peaks at the downstream of the gene. The far downstream Nrf2 peak contains one conserved Nrf2 element, GACTCA, which is complement to the Nrf2 core element, TGAGTC. The proximal downstream Nrf2 peak contains two conserved Nrf2 elements (Figure 4A, right panel). Thus, these data clearly indicated an Nrf2 dependency of the As^3+^-induced ATF6 and ATF6B.

To confirm the importance of Nrf2 in As^3+^-induced ATF6 and ATF6B, we also checked the mRNA expression of ATF6 and ATF6B between wild type (WT) BEAS-2B cells and the Nrf2 knockout (KO) cells that were made through CRISPR-Cas9 gene editing [22]. Corroborating to the ChIP-seq findings, the RNA-sequencing (RNA-seq) showed a notable decrease in both ATF6 and ATF6B in the Nrf2 KO cells relative to the WT cells (Figure 4B). Thus, we can conclude that Nrf2 plays a critical role in mediating As^3+^-induced expression of both ATF6 and ATF6B.

### 3.5. Contribution of Nrf2 to Other As^3+^-Induced UPR Genes

**Figure 5:**
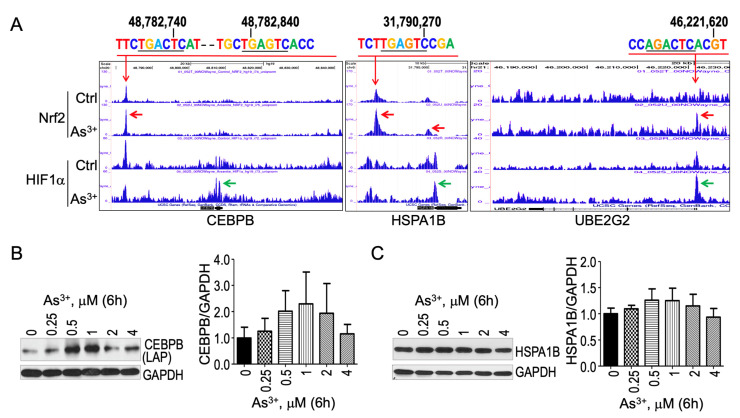
Nrf2 contributes to As^3+^-induced expression of CEBPB, HSPA1B and UBE2G2 (**A**). Screenshot for the indicated genes in ChIP-seq showing As^3+^-induced enhanced enrichment of Nrf2 (pointed by red arrows) and HIF1a (pointed by green arrows) (**B**,**C**). Western blotting and quantification of the Western blotting data from 3 independent experiments for CEBPB (**B**) and HSPA1B (**C**).

In addition to ATF6 and ATF6B, a real-time RT^2^ profiling of human UPR PCR array also indicated a strong induction of CEBPB, HSPA1B, MAPK10, and UBE2G2 (Figure 1). Analysis of the ChIP-seq data revealed that except MAPK10, all other genes, including CEBPB, HSPA1B and UBE2G2, exhibited an increased enrichment of Nrf2 binding on these gene loci in cellular response to As^3+^ (Figure 5A). CEBPB is a single exon gene at the locus of chr20 (q13.13). At 24.4 kb upstream of the CEBPB gene, there is a conserved Nrf2 peak that contains two Nrf2-binding elements with the core motif of TGACTC and TGACTC. In addition to Nrf2, As^3+^ also induced an enhanced enrichment of HIF1a at the gene body of CEBPB (pointed by a green arrow in Figure 5A). HSPA1B is also a single exon gene at the gene locus of chr6 (p21.33). There are two As^3+^-induced Nrf2 enrichment peaks, one is at the promoter region, and another one is about 5.2 kb upstream of the HSPA1B gene. A conserved Nrf2-binding element was found in the upstream peak (Figure 5A). An enhanced HIF1a enrichment by As^3+^ was noted at the promoter region of HSPA1B. As^3+^ is also highly capable of elevating the binding of Nrf2 and HIF1a at the promoter region of the UBE2G2 gene (Figure 5A, right panel). The notion that Nrf2 contributes to the As^3+^-induced expression of CEBPB, HSPA1B and UBE2G2 was supported by the dose-dependent increase in these proteins in the cells treated with As^3+^ (Figure 2D and Figure 5B).

### 3.6. Regulation of As^3+^ on Glycolysis and Mitochondrial Respiration

**Figure 6:**
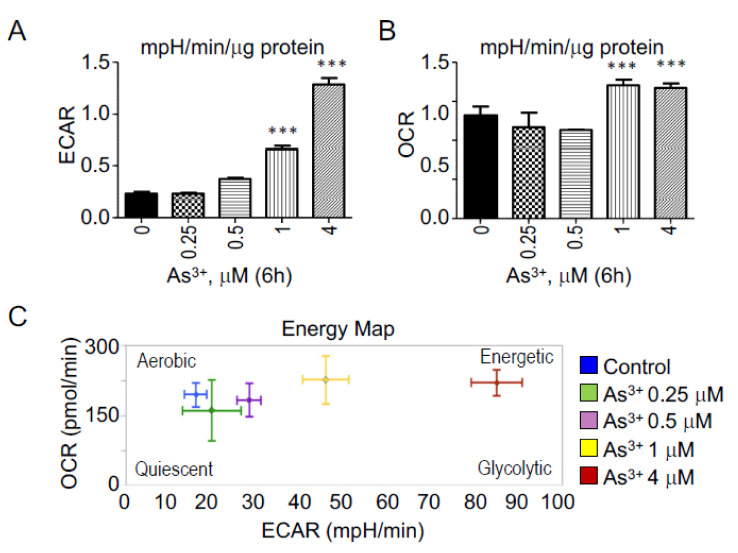
Real-time metabolic phenotype assay in As^3+^-treated BEAS-2B cells using Seahorse bioanalyzer (**A**). A significant upregulation was observed in the extracellular acidification rate (ECAR), especially at 1 μM and 4 μM As^3+^-treated BEAS-2B cells for 6h compared to control cells. Increased ECAR (mpH/min/mg protein) is indicative of active glycolytic metabolism (**B**). A significant upregulation was also observed in the oxygen consumption rate (OCR) at 1 μM and 4 μM As^3+^ doses in BEAS-2B cells. OCR (pmol/min) represents the mitochondrial respiration in the live cells (**C**). The ECAR versus OCR energy map shows that the BEAS-2B cells treated with As^3+^ shift towards a more energetic, glycolytic phenotype. *** *p* < 0.001.

The connection of ER stress to metabolic reprogramming had been well-established [22]. To determine the changes in the metabolic profile, the BEAS-2B cells were treated with different doses of As^3+^ for 6 h. The extracellular acidification rate (ECAR) and oxygen consumption rate (OCR) were measured using a Seahorse bioanalyzer. The ECAR and OCR values are indicators of glycolysis and mitochondrial respiration, respectively. A significant dose-dependent increase was observed in ECAR (Figure 6A) and OCR (Figure 6B) with As^3+^ treatment in BEAS-2B cells. The BEAS-2B cells seem to go towards a more energetic, glycolytic pathway with such a short-term As^3+^ treatment (Figure 6C). However, unlike in the As^3+^-induced CSCs and transformed cells, the short-term As^3+^ treatment also significantly increases mitochondrial oxidative phosphorylation.

### 3.7. Expression Status of ATF6 and ATF6B in Human Lung Adenocarcinoma

Considering the fact that As^3+^ is a human carcinogen and As^3+^ induces ATF6 and ATF6B, we next evaluated expression of ATF6 proteins in human lung adenocarcinoma tissues that were case-matched with the adjacent normal lung tissues using human tissue microarray (TMA) panels (LC10013c, US Biomax Inc. Rockville, MD, USA). We scored cancer and matched non-cancerous lung tissues into negative (1), weak positive (2), medium positive (3) and strong positive (4) based on visual staining intensity (Supplement Figure S1). We observed that of the 48 patients’ samples (cancer and matched non-cancer tissue pair), the ATF6 expression was higher in non-cancer tissue of 22 cases compared to their cancer tissue, whereas 15 cases had no difference and 11 cases had higher expression of ATF6 in cancer tissue compared to their non-cancerous tissue. Similarly, the ATF6B expression was higher in the non-cancer tissue of 28 participants compared to their non-cancerous tissue, whereas 11 cases had no difference and 9 cases had higher expression of ATF6B in cancer tissue compared to their non-cancerous tissue. No significant differences were observed in ATF6 and ATF6B among the cancer tissues stratified by grade, stage, age, and sex (data not shown).

### 3.8. The Prognostic Predictions of ATF6 and ATF6B Expression in Lung cancer Patients

**Figure 7:**
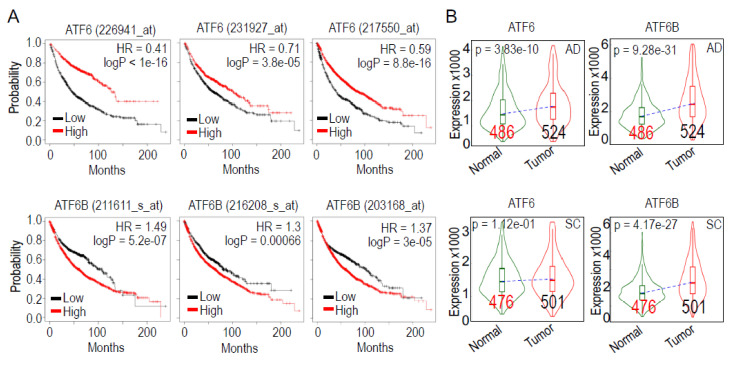
Different prognostic values of ATF6 and ATF6B for lung cancer patients (**A**). Data are derived from Kaplan–Meier database containing gene expression information from 1925 lung cancer patients. Probe IDs for ATF6 and ATF6B are indicated on the top of each panel. Analytic options of “Auto select best cutoff” for overall survival (OS) was used in graphic generation (**B**). Comparison of ATF6 and ATF6B gene in normal lung and tumor tissues in human lung adenocarcinoma (AD, top panels) and lung squamous cell carcinoma (SC, bottom panels) patients using the TNMplot. In AD, both ATF6 and ATF6B is significantly increased compared to normal lung tissues. In SC, only ATF6B was found to be significantly increased in the tumor tissues. Numbers in each column are total cases included in this analysis.

We have detected the expression of ATF6 and ATF6B in both normal lung tissues and lung cancer tissues. To additionally investigate whether the ATF6 genes contribute to the pathogenesis of human lung cancer, we evaluated the prognostic value of ATF6 expression on overall survival (OS) of lung cancer patients using the publicly available dataset from Kaplan–Meier Plotter [13]. We randomly selected 3 probe sets for both ATF6 and ATF6B (Figure 7A). By setting the analytic option of the best perform threshold as a cutoff, it showed that high level of ATF6 expression predicts a better OS of the lung cancer patients as determined by three different ATF6 probes (upper panels of Figure 7A). In contrast, the high level of ATF6B predicts a poorer OS for the patients (bottom panels of Figure 7A). The differences in the prognostic values between ATF6 and ATF6B are also reflected by the different expression levels of these two ATF6 genes between normal lung tissue and tumor tissues. In both lung adenocarcinoma (AD) and lung squamous carcer (SC), the tumors expressed much higher levels of ATF6B relative to ATF6 (Figure 7B). These data, thus, suggested possible different roles played by ATF6 and ATF6B in the development of human lung cancer, despite the fact that ATF6 and ATF6B can form heterodimers for transcriptional regulation. ATF6 may more likely tend to be a tumor suppressor, whereas ATF6B appears to be more oncogenic.

## 4. Discussion

Arsenic, derived from the Greek word “arsenikos,” meaning potent, is a metalloid naturally present in the earth’s crust. However, natural and predominantly anthropogenic activities such as mining and agriculture have disturbed arsenic’s geochemical cycles, leading to its exposure in food, water, and air [23]. Various case–control, cohort and ecological studies have demonstrated a positive correlation between high arsenic exposure and lung cancer development [24]. To protect human health, the WHO International Agency for Research on Cancer (IARC) and US Environmental Protection Agency (EPA) have set an arsenic limit of 10 ppb in drinking water. Despite the regulations, arsenic exposure, especially the inorganic trivalent arsenic (As^3+^) via contaminated drinking water is still a global health issue affecting more than 200 million people worldwide [12].

Previous studies from our group have shown that long-term treatment of the BEAS-2B cells with the environmentally relevant concentration of As^3+^ leads to the malignant transformation of these cells, and some of these cells acquire cancer stem-like (CSC) properties. Gene profiling and metabolomics analysis suggest impairment of the ER stress signaling, autophagy and mitochondrial functions [7]. It is well documented that the UPR, mitochondria and autophagy work in conjunction under internal and external cellular stresses. It is also well known that the tri-components of the global ER stress response can restore cellular homeostasis or activate apoptosis under unresolved stress conditions [12,25]. However, since the long-term treatment procedure very likely causes adaptation and repopulation of the cells, it is important to determine whether the altered ER stress signaling is an initial event or a result of cellular adaptation. In a real-time metabolic phenotype assay in BEAS-2B cells treated with As^3+^ for 6 h compared to control cells, we indeed noted a significant upregulation in glycolysis at a relatively high concentration of As^3+^ (Figure 6A), which is consistent with our results in As^3+^-induced CSCs [7]. This finding is also in agreement with the report by Zhao et al., who showed that As^3+^ exposure in BEAS-2B cells induced an increased rate of extracellular acidification, which was inhibited by non-metabolized glucose analog 2-deoxy-D-glucose [26]. Interestingly, unlike what was observed in the CSCs, the short-term As^3+^ treatment also upregulated the mitochondrial respiration rates (Figure 6B). Such a difference in mitochondrial metabolism between short- and long-term As^3+^ treatment is very likely due to the accumulative damages of the functional proteins in mitochondria during a long-term As^3+^ exposure.

Treatment of the BEAS-2B cells with As^3+^ also caused a more than two-fold upregulation of five UPR genes, including ATF6, CEBPB, HSPA1B, MAPK10 and UBE2G2. ATF6 is an ER-resident UPR protein and a member of the basic leucine zipper family of transcription factors. It is a 90 kDa protein that is cleaved to a 50 kDa protein in the Golgi apparatus under ER stress. The cleaved form of N-ATF6 translocates to the nucleus to activate ER stress response genes [27]. Mammals express two isoforms of ATF6 proteins, the bona fide ATF6, ATF6A (670 aa), and ATF6B (703 aa). The ATF6 and ATF6B proteins have similar highly conserved b-Zip domains that allow them to bind to ER stress response element (ERSE) as homo or heterodimers. However, they have a divergent N-terminal domain and vary in their ability to induce an ER stress response. The ATF6 protein is more studied than ATF6B protein [20]. In the present study, we found that both ATF6 and ATF6B protein levels can be induced by As^3+^, and both proteins showed proteolytic products in the As^3+^-treated cells (Figure 2 and Figure 3) in the As^3+^-treated cells. It has been implicated that only the cleaved forms of ATF6 and ATF6B can function as transcription factors for the transcription of the ER stress response genes in human bronchial epithelial cells, such as Grp78, Grp94, Xbp1, VEGF, etc. [28,29].

Mutual regulation of ER stress and Nrf2 activation had been studies in *C. elegans* and certain neurodegenerative diseases [30]. In this report, we provided the first evidence showing that Nrf2 is also a master regulator for the expression of the ER stress-associated UPR in cellular response to As^3+^ through ChIP-seq and RNA-seq. On the genomic level, all of the As^3+^-induced UPR genes contain conserved Nrf2-binding elements in either the promoter region or upstream of the transcription start site. Furthermore, some of them also showed significant enrichment of HIF1a binding in the promoter or gene body, such as CEBPB, HSPA1B and UBE2G2. HIF1a is a well-established oncogenic transcription factor during tumorigenesis. We had shown that the non-hypoxic induction of HIF1a by As^3+^ is also Nrf2 dependent [22]. Accordingly, there is a self-amplification and forward feedback loop among ER stress, Nrf2 and HIF1a induced by As^3+^, which may serve as one of the key mechanisms of As^3+^-induced carcinogenesis.

Although IHC evaluation of the ATF6 and ATF6B in lung cancer tissues vs. case-matched non-cancerous tissues is somewhat inconclusive, the overall survival analysis for the lung cancer patients unraveled an opposite prognostic value between ATF6 and ATF6B. Higher expression of ATF6 and ATF6B predicts a better and poorer OS of the lung cancer patients, respectively, indicating a possible tumor suppressor-like activity of ATF6 and an oncogenic property of the ATF6B for lung carcinogenesis. These findings also suggest that ATF6 and ATF6B may have different target genes for transcriptional regulation under physiological and carcinogenic conditions. Future studies are needed to define what sets of genes are regulated by these two transcription factors. Taken together, the data presented in this report demonstrated that As^3+^ is capable of activating the ER stress response signaling that may be actively involved in mediating the carcinogenic and/or tumorigenic processes associated with environmental As^3+^ exposure.

## Data Availability

All ChIP-seq data can be accessed through NCBI Gene Expression Omnibus.

## Supplementary Materials

The following supporting information can be downloaded at https://www.mdpi.com/article/10.3390/biomedicines10050967/s1. Figure S1: ATF6 and ATF6B protein expression in lung adenocarcinoma and matched non-cancer tissues from patients.
