# Supplementary material for: Arsenic Activates the ER Stress-Associated Unfolded Protein Response via the Activating Transcription Factor 6 in Human Bronchial Epithelial Cells
Source: Biomedicines. 2022 Apr 22;10(5):967. doi: 10.3390/biomedicines10050967 (PMC9139116; doi:10.3390/biomedicines10050967)
Supplement: Supplementary file 1 [file biomedicines-10-00967-s001.zip › biomedicines-1660586-supplementary.pdf]

Supplement Figure S1. ATF6 and ATF6B protein expression in lung adenocarcinoma and matched non-cancer tissues from patients. IHC staining scores are negative (1), weak positive (2), medium positive (3), strong positive (4). **A.** Representative immunohistochemistry images of ATF6 and ATF6B in lung adenocarcinoma and the case-matched non-cancerous lung tissues of 48 patients. **B.** Quantification summary of the ATF6 (top panel) and ATF6B (bottom panel) status cancer and non-cancer tissues of 48 patients.

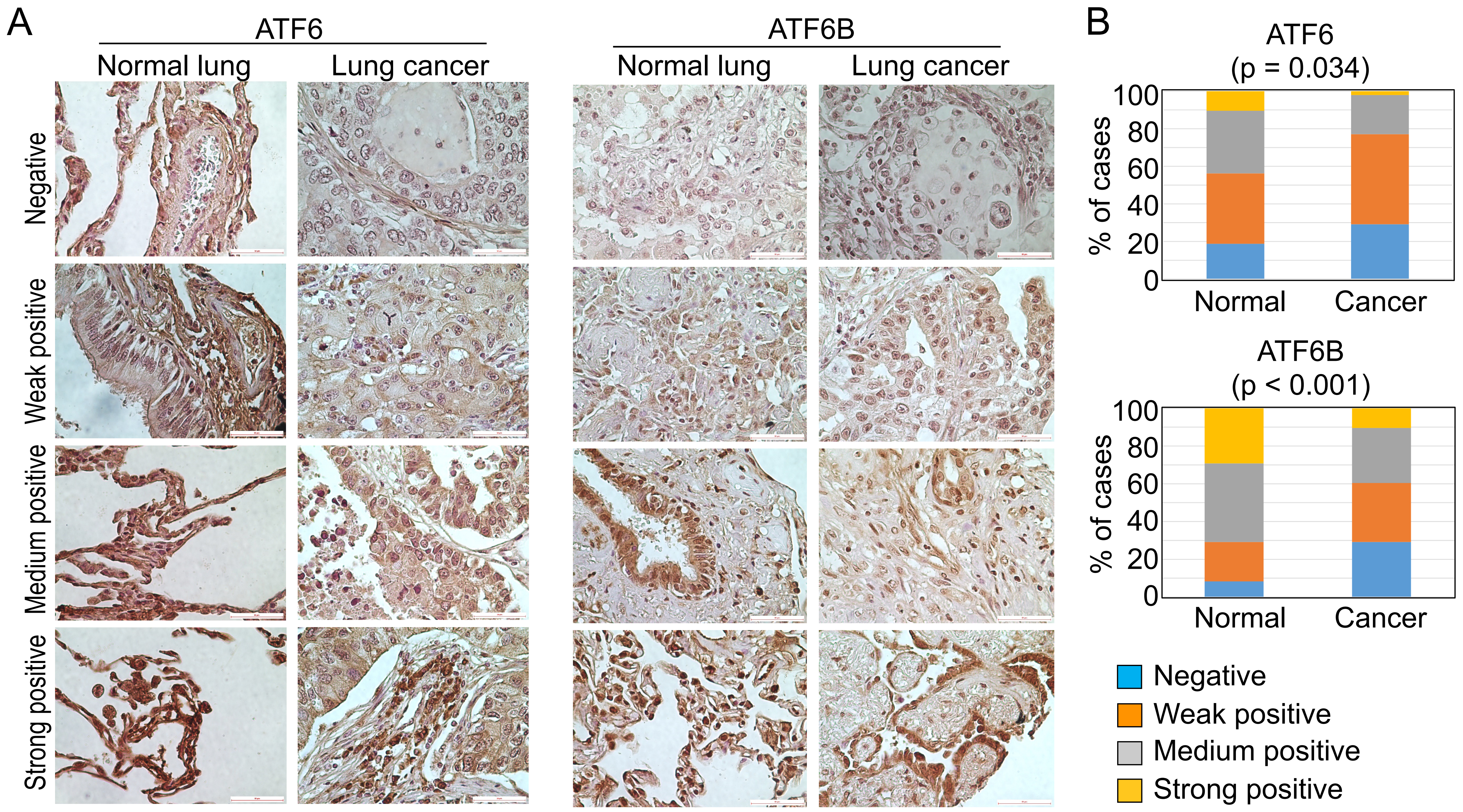
A
ATF6
Normal lung
Lung cancer
ATF6B
Normal lung
Lung cancer
Negative
Weak positive
Medium positive
Strong positive
B
ATF6
(p = 0.034)
% of cases
100
80
60
40
20
0
Normal
Cancer
ATF6B
(p < 0.001)
% of cases
100
80
60
40
20
0
Normal
Cancer
Negative
Weak positive
Medium positive
Strong positive
